# Critical care nurses’ communication experiences with patients and families in an intensive care unit: A qualitative study

**DOI:** 10.1371/journal.pone.0235694

**Published:** 2020-07-09

**Authors:** Hye Jin Yoo, Oak Bun Lim, Jae Lan Shim

**Affiliations:** 1 Department of Nursing, Asan Medical Center, Seoul, South Korea; 2 College of Medicine, Department of Nursing, Dongguk University, Gyeongju, South Korea; Victoria University, AUSTRALIA

## Abstract

This study evaluated the communication experiences of critical care nurses while caring for patients in an intensive care unit setting. We have collected qualitative data from 16 critical care nurses working in the intensive care unit of a tertiary hospital in Seoul, Korea, through two focus-group discussions and four in-depth individual interviews. All interviews were recorded and transcribed verbatim, and data were analyzed using the Colaizzi’s method. Three themes of nurses’ communication experiences were identified: facing unexpected communication difficulties, learning through trial and error, and recognizing communication experiences as being essential for care. Nurses recognized that communication is essential for quality care. Our findings indicate that critical care nurses should continuously aim to improve their existing skills regarding communication with patients and their care givers and acquire new communication skills to aid patient care.

## Introduction

Critical care nurses working in intensive care units (ICUs) care for critically-ill patients, and their work scope can include communicating with patients’ loved ones and care givers [1]. In such settings, nurses must make timely judgments based on their expertise, and this requires a high level of communication competency to comprehensively evaluate the needs of patients and their families [2,3]. The objective of nurses’ communication is to optimize the care provided to patients [4]. Therapeutic communication, a fundamental component of nursing, involves the use of specific strategies to encourage patients to express feelings and ideas and to convey acceptance and respect. In building an effective therapeutic relationship, a focus on the patient and a genuine display of empathy is required [5]. Empathy is the ability to understand and share another person’s emotions. To convey empathy towards a patient, one must accurately perceive the patient’s situation, communicate that perception to the patient, and act on the perception to help the patient [6]. Effective communication based on empathy not only contributes greatly to the patient's recovery [3,5–7], but also has a positive effect of improving job satisfaction by nursing with confidence [8] In contrast, inefficient communication leads to complaints and anxiety in patients and can also lead to other negative outcomes, such as extended hospital stays, increased mortality, burnout, job stress, and turnover [9,10].

Therefore, communication experiences need investigation since effective communication is an essential for critical care nurses. Nurses use curative communication skills to provide new information, encourage understanding of patient’s responses to health troubles, explore choices for care, help in decision making, and facilitate patient wellbeing [11]. Particularly, patient- and family-centered communication contributes to promoting patient safety and improving the quality of care [12,13]. However, communication skills are relatively poorly developed among critical care nurses compared to nurses in wards and younger and less experienced nurses than in their older and more experienced counterparts [3,7,14–16]. This calls for an examination of the overall communication experiences of critical care nurses.

To date, most studies on the communication of critical care nurses have been quantitative and have evaluated work performance, association with burnout, and factors that hinder communication [2–4,7]. A qualitative study has examined communications with families of ICU patients in Korea [17], while an international study has identified factors that promote or hinder communication between nurses and families of ICU patients [16,18]; however, few studies have been conducted on participant-oriented communication (including patients and families). Nurses’ communication with patients and their families in a clinical setting is complex and cannot be understood solely on the basis of questionnaire surveys; therefore, these communication experiences must be studied in depth.

This study explored critical care nurses’ communication experiences with patients and their families using an in-depth qualitative research methodology. This study will help to enhance communication skills of critical care nurses, thereby improving the quality of care in an ICU setting.

## Materials and methods

### Design

This study employed a qualitative descriptive design using focus-group interviews (FGIs) and in-depth individual interviews and was performed according to the consolidated criteria for reporting qualitative research (COREQ) checklist [19]. An FGI is a research methodology in which individuals engage in an intensive and in-depth discussion of a specific topic to explore their experiences and identify common themes based on the interactions among group members [20]. Individual in-depth interviews were also conducted to complement the content identified in FGIs and further explore the deeper information developed based on experiences at the individual level.

### Participants

Sixteen critical care trained nurses providing direct care to patients in an ICU of a tertiary hospital in Seoul were included in this study. The purpose of this study and the contents of the questionnaire were explained to them, and they voluntarily agreed to participate and complete the questionnaire. The exclusion criteria were as follows: 1) nurses with a hearing problem; 2) nurses with less than 1 year of clinical experience; and 3) nurses diagnosed with psychiatric disorders.

Snowball sampling—in which participants recruit other participants who can vividly share their experiences regarding the topic under investigation—was used. Six participants for the first FGI, six for the second FGI, and four for the individual in-depth interviews were recruited. All participants were women (mean age = 29.0 years old; mean nursing experience = 4.5 years). Their characteristics are listed in Table 1.

**Table 1:** Participant characteristics.

No.	Sex	Age (years old)	Intensive care unit experience (months)	Marital status	Highest Educational Level
1	F	28	30	Single	University
2	F	27	30	Single	University
3	F	27	29	Single	University
4	F	29	27	Single	University
5	F	27	24	Single	University
6	F	26	24	Single	University
7	F	26	22	Single	University
8	F	26	22	Single	University
9	F	26	22	Single	University
10	F	26	20	Single	University
11	F	27	20	Single	University
12	F	26	20	Single	University
13	F	29	40	Single	University
14	F	37	168	Married	Master’s
15	F	38	180	Married	Master’s
16	F	39	188	Married	Master’s

### Data collection

#### Developing interview questions

The interview questions were structured according to the guidelines developed for the focus-group methodology [21]: 1) introductory questions, 2) transitional questions, 3) key questions, and 4) ending questions. The questions were reviewed by a nursing professor with extensive experience in qualitative research and three critical care nurses with more than 10 years of ICU experience (Table 2). These questions were also used for individual face-to-face in-depth interviews.

**Table 2:** List of interview questions.

Question Type	Questions
Introductory	What kind of care do you provide to your patients and their families as an ICU nurse?
Transitional	As an ICU nurse, how is your communication with your family now?
Key	What is your primary focus when communicating with patients and their families? Do you have memorable experiences in your communication with your patients’ families? a) If so, what were these experiences? b) How do you feel about those experiences? Do you have your own strengths in communicating with patients and their families? a) If so, what are their advantages? b) What role do your strengths play in communication? c) What is the impact of communication on nursing? Have you ever faced difficulties in communicating with patients’ families? a) If so, please specify them. b) What is the impact of these communication difficulties on your patients and their families? c) How do these communication difficulties affect nursing? Have you made any personal effort to communicate effectively with patients and their families? a) If so, what have you done specifically? b) How does the effort/s you have made affect your communication with patients and their families? Do you need hospital or external help to improve communication with your patients and their families? a) If so, what specific help do you need? b) How do you feel about the changes in communication style with patients and families when support and help are provided? What does communication with the patients and their families mean to the nurse?
Ending	Is there anything you would like to add?

#### Conducting FGIs and individual interviews

The two FGIs and four individual interviews were conducted between July 20, 2019 and September 30, 2019. The FGIs were moderated by the principal female investigator and were conducted in a quiet conference room where participants were gathered around a table to encourage them to talk freely. The FGIs were audio-recorded with the participants’ consent, and the recordings were transcribed and analyzed immediately after. Similar content was observed from the two rounds of FGIs, and we continued the discussion until no new topics emerged.

To complement the FGIs and verify the results of the analysis, we also conducted individual interviews of four participants. One assistant helped in facilitating the interviews and took notes. The duration of each interview was about 60–90 minutes.

#### Ethical considerations and investigator training and preparation

This study was approved by the institutional review board of the Asan Medical Center (approval no. 2019–0859). Prior to data collection, participants provided written informed consent and were informed in advance that the interviews would be audio-recorded, their participation would remain confidential, the recordings and transcripts would only be used for research purposes, the data would be securely stored under a double lock and would be accessed by the investigators only, and personal information would be deleted upon the completion of the study to eliminate any possibility of a privacy breach. The collected data were coded and stored to be accessed by the investigators only to prevent leakage of any personal information.

The authors of this study are nurses with more than 10 years of ICU experience and a deep understanding of critical care. The principal investigator took a qualitative research course in graduate school and has conducted multiple qualitative studies to enhance her qualitative research experience.

### Data analysis

We utilized Colaizzi’s [22] method of phenomenological analysis to understand and describe the fundamentals and meaning of nurses’ communication experiences with patients and families. Data analysis was conducted in seven steps: 1) Recording and transcription of the in-depth interviews (all authors read the transcripts repeatedly to better understand the participants’ meaning); 2) Collection of meaningful statements from phrases and sentences containing phenomena relating to the communication experiences in the ICU. We extracted statements overlapping with statements of similar meaning—taking representative ones of similar statements—and omitted the rest; 3) Searching for other interpretations of participant statements using various contexts; 4) Extraction of themes from relevant meanings and development of a coding tree, with the meanings organized into themes; 5) Organization of similar topics into a more general and abstract collection of themes; 6) Validation of the collection of themes by cross-checking and comparing with the original data; 7) After integrating the analyzed content into one technique, the overall structure of the findings was described.

During data analysis, we received advice on the use of language or result of analyzing from a nursing professor with extensive experience in qualitative research and had the data verified by three participants to establish the universality and validity of the identified themes.

### Establishing precision

The credibility, fittingness, auditability, and confirmability of the study were evaluated to analyze our findings [23]. To increase credibility, we conducted the interviews in a quiet place to focus on participants’ statements and help participants feel comfortable during interviews; to establish the universality and validity of the identified themes, data verification was performed by three participants. To ensure uniformity in data, participants who could provide detailed accounts of their experiences were selected, and the data were collected and analyzed until saturation was achieved (i.e., no new content emerged). To ensure auditability, the raw data for the identified themes were presented in the results, such that the readers could understand the decision-making process. To ensure confirmability, our preconceptions or biases regarding the participants’ statements were consistently reviewed to minimize the impact of bias and maintain neutrality.

## Results

After analyzing the communication experiences of 16 critical care nurses, three major themes emerged: facing unexpected communication difficulties, learning through trial and error, and recognizing communication experiences as being essential for care. The results are summarized in Table 3.

**Table 3:** Critical care nurses’ communication experience with patients and their families.

Sub-category	Category	Theme
		Theme 1: Facing unexpected communication difficulties
In critical care, communication with patients and their family is burdensome	1.1. True intentions not conveyed as wished	Nurse-related
Misunderstanding because of the linguistic characteristics of a nurse (e.g., dialect, voice tone, etc.)		
Impatience/lack of care for patients and caregivers		
ICU nurses need both verbal and nonverbal communication skills	1.2. Hesitant to provide physical comfort	
Nonverbal communication is unfamiliar		
Not sure how to effectively provide nonverbal communication		
Patient in ventilator feels frustrated because he or she cannot speak	1.3. Mechanical ventilation hindering communication with the patient	Patient- and family-related
Difficulty understanding because the patient is on a ventilator and thus cannot speak		
Ventilator interferes with the communication between nurse and patient		
ICU patient’s caregiver is sensitive	1.4. Caregivers’ negative responses to nurses	
Normal communication is impossible owing to caregivers’ aggressive attitude		
As an ICU nurse, I have no choice but to respond to the conversation		
I have not learned properly about communication in the clinic	1.5. Lack of experience and a mismatch between theory and practice	System-related
Communication is the most difficult task for less experienced, young nurses		
The scheduled visit time in the intensive care unit is when most communication occurs	1.6. Intense visiting hours in limited time	
One-way conversation with the caregiver in a short period		
Life-dependent care is a priority in the intensive care unit	1.7. Urgent workplace that deprioritizes communication	
Insufficient time to talk with patients and caregivers owing to heavy workload		
Nurses are hurt by distrustful patients and caregivers	2.1. Fundamental doubts about the nursing profession	Theme 2: Learning through trial and error
Difficulty in nursing because of trauma from patients and caregivers		
Follow senior nurses and learn practical communication	2.2. Finding out which communication style is better suited for patients and their families	
Explains the patient's daily life in detail		
Communication is indispensable to nursing	2.3. Knowhow learned through persistent effort	
Studying the lack of communication by searching books and videos		
Understand the anxiety and difficulties experienced by the critically ill and their caregivers	3.1. Empathy garnered through various clinical experiences	Theme 3: Recognizing communication experiences as being essential for care
Nurse's words have the power to make the patients and their families cry or laugh		
Listening as an intensive care nurse is more important than talking	3.2. The power of active listening	
Nurse empathy strengthens patients and caregivers and enhances their feelings of control		
Patients and caregivers are easy to reach	3.3. Mediator between physicians, patients, and caregivers	
Nurses use words that are easy to understand		
Nurses convey sincerity to others with respect and understanding	3.4. Expressing warmth and respect	
Nurses’ heartfelt expressions promote trust		

**Fig 1:**
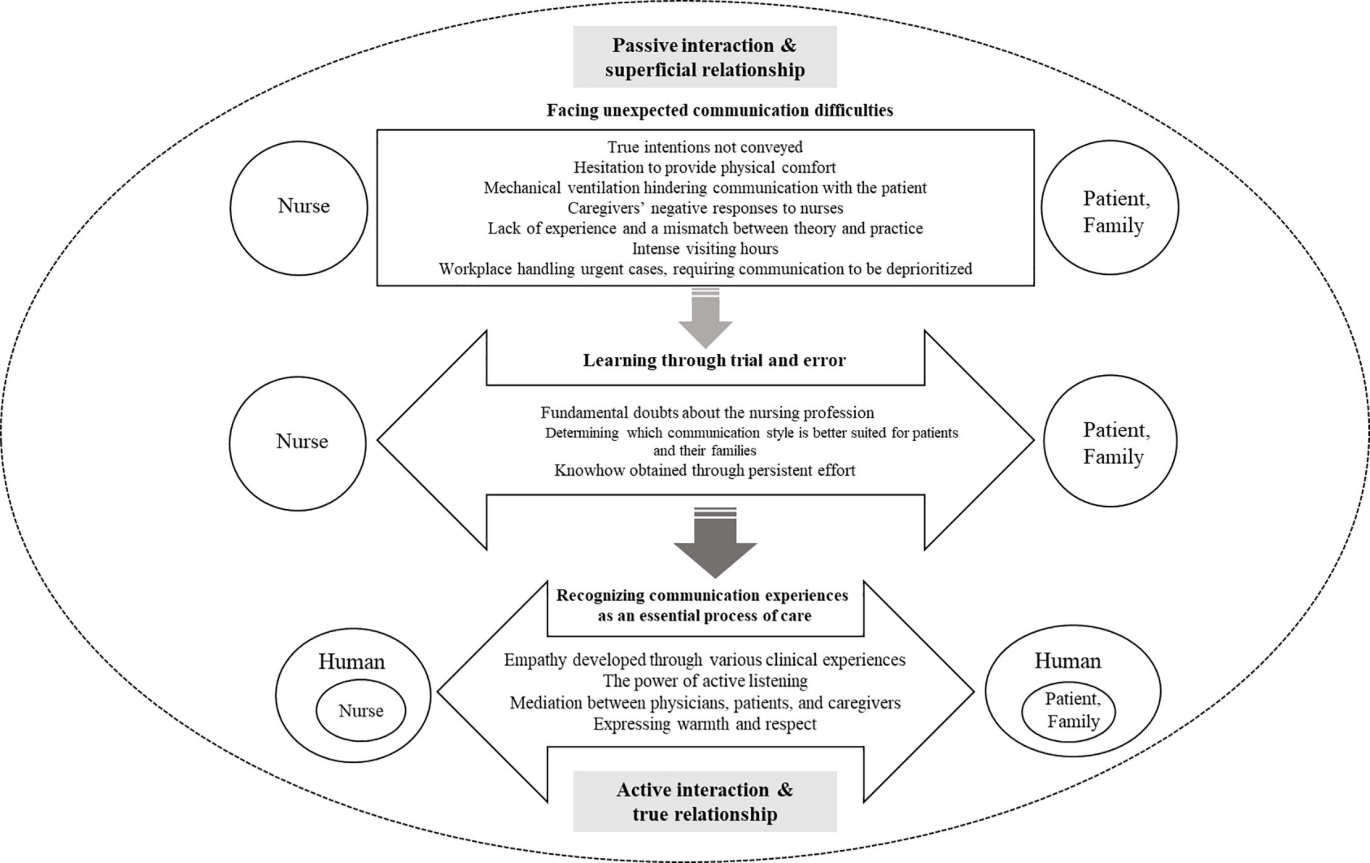
Summary of communication experiences encountered by intensive care unit nurses.

The results of this study are schematized based on Travelbee's Human-to-Human Relationship Model [24,25] (Fig 1), which suggests that human-to-human interaction is at a developmental stage. In this study, communication between patients and their families and experienced nurses in ICUs promotes human-to-human connections, leading to a genuine caring relationship through interaction, empathy, listening, sharing, and respect, which are all therapeutic communication skills.

### Theme 1: Facing unexpected communication difficulties

Nurses experienced more difficulties in communicating with patients and their families and caregivers than in performing essential nursing activities (e.g., medication administration, suction, and various mechanical operations) The communication difficulties they experienced were either nurse-, patient- and family-, or system-related. Distinct problems in an ICU are related to urgency; for example, hemodynamically unstable patients or patients with respiratory failure or those suffering from a cardiac arrest may be prioritized.

#### Nurse-related factor: True intentions were not conveyed as wished

Although nurses intend to treat patients and their families with empathy, they frequently lead one-way conversations when pressed for time in the ICU. In addition, their usual way of talking, such as their dialect and intonation, can sometimes be misunderstood and cause offense. Participants experienced difficulties communicating their sincerity to patients and their families.

“Oftentimes, I only say what I have to say instead of what the caregivers really want to know when I’m pressed for time to convey my thoughts and go on to the next patient to explain things to the other patient.” (Participant 2)

“I usually speak loudly, and I speak in dialect; so, things I say are not delivered gently…I try to be careful because my dialect can seem more aggressive than the Seoul dialect; but it’s not easy to fix what I have used for all my life at once.” (Participant 3)

#### Nurse-related factor: Hesitant to provide physical comfort

Participants were not familiar with using non-verbal communication. The participants realized the importance of both verbal communication and physical contact in providing care, but the application of both these communication styles was not easy in clinical practice.

“I want to console the caregivers of patients who pass away; but I just can’t because I get shy. I feel like I’m overstepping, and when I’m contemplating whether I can really speak to their emotions, the caregiver has already left the ICU in many cases.” (Participant 6)

“I’m really bad at physical contact even with my close friends; but I’m even worse when it comes to patients and caregivers. Because of my tendency, there are times when I hesitate to touch patients while providing care.” (Participant 7)

#### Patient- and family-related factor: Mechanical ventilation hindering communication with the patient

Mechanical ventilators were the greatest obstruction to communication in ICU. Although it is normal for patients on a mechanical ventilator to lose the ability to speak, patients and their families did not understand how mechanical ventilators work and were frustrated that they could not communicate freely with the patient. Participants expressed difficulty in communicating with patients in ways other than verbal communication as well.

“Patients who are on mechanical ventilation can’t talk as they want and do not have enough strength in their hands to write correctly; so, even if I try to listen to them, I just can’t understand what they are saying. You know in that game where people wear headphones playing loud music and try to communicate with one another—words completely deviant from the original word are conveyed. It just feels like that.” (Participant 9)

“Patients on mechanical ventilation and who thus cannot communicate are the most difficult. The patient keeps talking; so, it hinders respiration—the ventilator alarm keeps going off, the stomach becomes gassy, and the patient has to take the tube off and vomit later. No matter how much I explain, there are patients or caregivers who tell me that the tube in the throat is making [it] hard [for them] to breathe or [they] ask me to take it off just once and put it back on, and these patients are really difficult. There is no way to communicate if they cannot accept mechanical ventilators even if I explain.” (Participant 8)

#### Patient- and family-related factor: Caregivers’ negative responses to nurses

It was also burdensome for nurses to communicate with extremely stressed caregivers and loved ones, especially when patients were in a critical state. Despite the role of nurses in helping patients during health recovery, caregivers’ negative responses to nurses, such as blaming them and speaking and behaving aggressively, intimidated the participants and ultimately discouraged conversations.

“I can manage the patients’ poor vital signs by working hard but communicating with sensitive caregivers who project their anxiety about the patient’s state onto nurses doesn’t go as I wish, so, it’s really difficult and burdensome.” (Participant 6)

“When the patient is in a bad state, caregivers sometimes want to not accept it and project their feelings onto the nurses, and in such cases, there are no words that can console them. Even approaching the caregivers is a burden, and I get kind of intimidated.” (Participant 5)

#### System-related factor: Lack of experience and a mismatch between theory and practice

Participants have learned the importance of communication during training; however, they had trouble appropriately applying the learned concepts in their workplace. Participants in this study were in their 20s and 30s, with limited life and social experiences, and felt the gap between theory and practice in communicating with patients and families in ICU.

“Talking to the patient or caregiver was the most challenging thing when I was new…it is impossible for nurses with not much life experience to communicate skillfully.” (Participant 10)

“It would be nice if the real-world conversation proceeds in the way shown in our textbook; but it doesn’t in most cases. So, it is more practical to observe and learn from what other nurses do in the actual field.” (Participant 2)

#### System-related factor: Intense visiting hours in limited time

The 30-minute ICU visiting period is the only time when patients and families can talk to one another. Although nurses are well trained to care for the patients to the best of their ability, caregivers distrust the nurses’ ability to care for patients since caregivers only have a limited amount of visiting time, thus hindering effective communication. Some participants even experienced mental trauma following short but unforgettable interactions with caregivers.

“…the caregiver browbeat me and intimidated me for doing so. This gave me a mental trauma for visiting hours…I didn’t know how to start a conversation and the visiting hours were really stressful for me.” (Participant 3)

“The caregivers don’t stay in the ICU for 24 hours; so, once they begin to doubt our nursing practice, we cannot continue our conversation with them…” (Participant 11)

#### System-related factor: Urgent workplace that deprioritizes communication

The ICU is a unit for treating critically-ill patients; therefore, ICU nurses were more focused on tasks directly linked to maintaining patients’ health, such as stabilizing vital signs, than on communication. Participants frequently encountered emergency situations, in which they could not idly stay around to communicate with one patient because another required immediate assistance, i.e., they faced a reality in which they had to prioritize another patients’ health over communication with one.

“…I’m really pressed for time when the patient keeps writing things I can’t understand with their weak hands…I don’t have time to spare even if I want to listen to them.” (Participant 12)

“Vital signs are the utmost priority in [the] ICU. I’m on my feet the entire shift and don’t even have time to go to the restroom…During early ICU treatment, there are a lot of emergency situations; so, communication is way down in the priority list.” (Participant 5)

### Theme 2: Learning through trial and error

The negative experiences arising from communicating with various individuals sometimes forced nurses to think twice about their vocation; however, due to a sense of responsibility, they tried to engage in therapeutic communication and to overcome difficulties.

#### Fundamental doubts about the nursing profession

Experiencing unfriendly and confrontational conversations with patients and caregivers was intolerable for participants. These experiences were shocking enough to make them fundamentally question their decision to choose and stay in the nursing profession.

“I felt so disappointed and frustrated when patients or caregivers bombard[ed] rude comments at me with complete disregard of what I have done over a long period…I can’t sleep well at night and my values as [a] nurse are shaken from their root.” (Participant 14)

“It becomes so difficult the moment communication fails and mutual trust is lost. Maybe I could survive if this is just with one patient or caregiver; but the afterimage lingers with me persistently while I’m working…I came to think whether I could continue nursing.” (Participant 7)

#### Finding out which communication style is better suited for patients and their families

Nurses learned how to resolve communication-related difficulties that they encountered from their seniors and mentors and tried to communicate better from their position at the nursing station.

“A senior nurse of mine was talking to a caregiver who was really concerned, and she was using affirmations like ‘Oh, really’ and ‘I see’ with a relaxed facial expression, and the caregiver would spill her heart out to her. That’s when I thought that empathy is to express responses to what the other person is saying.” (Participant 10)

“I can feel that I am able to bond with patients’ families when I tell them about the patient’s daily living, such as how much the patient had slept, eaten, and whether the patient was not in pain, during visiting hours.” (Participant 13)

#### Knowhow learned through persistent effort

Nursing activities, such as taking vital signs and performing aspiration and intravenous injection, are learned over time; however, it is impossible to acquire therapeutic communication skills without personal effort and interactive experiences in the field.

“I’m reading a book about conversation and am learning about how to express empathy and understand other people…Nursing skills are developed and improved over time; but it’s not easy to enhance communication without personal effort or change in perception.” (Participant 16)

“Communication is an indispensable part of nursing. If you want to provide high-quality care, you need to enhance your communication skills first.” (Participant 15)

### Theme 3: Recognizing communication experiences as being essential for care

Nursing and communication are inseparable. Although communication is a challenge while caring for ICU patients, therapeutic communication is important for the patients’ and their families’ overall wellbeing. In an ICU, communication based on empathy and experience is a significant component that helps patients perceive their illnesses more positively.

#### Empathy garnered through various clinical experiences

Since participants met many patients and their families in the ICU, they were able to communicate. Participants understood patients’ discomfort and learn why it was difficult for them to communicate and to comfort and assure unease families who could not observe the patient's condition. However, it was a necessary communication method in the ICU. Participants realized the value and importance of their words.

“…his endotracheal tube was touching his throat and was so uncomfortable: his mouth was dry, he couldn’t talk, and his arms were tied; so, he thought the only way to communicate was to use his legs and that’s why he was kicking. I felt really sorry…” (Participant 7)

“I gave a little detailed explanation to the caregiver during visiting hours and she thanked me overwhelmingly. I feel that, because this is the ICU, patients and caregivers can be encouraged and discouraged by the words of the medical professionals.” (Participant 9)

#### The power of active listening

Although the ability to handle tasks promptly is important, listening to patients amid the hectic work schedule in the ICU is also an important nursing skill. Critical care nurses realized that listening to patients and caregivers without saying anything is also meaningful and therapeutic.

“I was listening to the caregiver the entire duration of the visiting hour…She said that she just had to open up to someone to talk about her frustrations, and that my listening to her was a huge consolation for her.” (Participant 12)

“While listening to the caregiver and showing empathy every day at the same time, I was able to witness that the caregiver who had been aggressive and edgy changed in a way to trust in and depend on the nurse more.” (Participant 16)

#### Mediator between physicians, patients, and caregivers

Participants were at the center of communication, serving as the bridge connecting physicians to patients and patients to caregivers. They served as mediators, explaining the doctors’ comments to the caregivers, and providing details regarding the patients’ state to families. Participants helped maintain a close and balanced relationship between the doctor, the patients, and their families by conveying messages not effectively communicated by the doctor or patients.

“Caregivers would not ask any questions to the doctor in the ICU and would ask me instead once the doctor is gone. They would ask, ‘what did the doctor say?’ and ask me for an explanation.” (Participant 4)

“The patients can’t say everything they want; so, as nurses, we are the mediators between patients and caregivers…Tell[ing] the family about things that happened when they were not around the patient is meaningful.” (Participant 14)

#### Expressing warmth and respect

Participants have experienced sharing emotions with the patient's family as well as with the patient during disease improvement and exacerbation.

In particular, sincere actions, such as staying with the families of patients who died or those whose condition was deteriorating, led to more genuine relationships, as respect for human life was expressed.

“When patients whom we have spent a long time [with] are about to pass away, we cry for them and we stay beside them in their final moments…Showing respect for a person’s final moments of life and expressing our hearts is meaningful, and it is something critical care nurses must do.” (Participant 16)

“When the patient’s state worsened and…his daughter was sobbing next to him…I softly touched her shoulder, and she really thanked me. As I saw the patient’s family grieve, I just expressed how I felt, and, fortunately, my intention was well conveyed” (Participant 4)

## Discussion

This study evaluated critical care nurses’ communication skills and experiences with patients and their caregivers. Based on the two FGIs and four individual in-depth interviews, three themes have been identified: 1) facing unexpected communication difficulties; 2) learning through trial and error; and 3) recognizing communication experiences as being essential for care

For theme 1, we examined nurse-, patient-, family-, and system-related (i.e., pertaining to hospital resources and education) factors. Theme 1 can be considered as the communication involving human-to-human interaction, as mentioned in Travelbee [24,25], that takes place at an incomplete stage. First, critical care nurses struggled with balancing their heavy workload and communicating with patients and their families. In Korea, an ICU nurse, on an average, cares for two to four patients, which is higher than in some other countries, wherein an ICU nurse cares for one or two patients at the most; thus, the Korean work environment for ICU nurses is more stressful [26]. This limits the amount of time nurses may have to communicate and interact with their patients and caregivers. Misunderstandings are also common owing to the patients’ inability to speak while intubated and to use of regional dialects. Patients and caregivers want to hear specific and comprehensible information from health professionals regarding the treatment procedures in the ICU [17,27]. However, previous studies [4,28] have reported that critical care nurses experience communication difficulties due to high mental pressure due to work, time constraints, and the inability to use their own language; these are consistent with our findings. As nurses are required to interact with patients having various needs, they need to learn how to communicate verbally and nonverbally in a sophisticated manner [27], and hospital managers should implement practical communication programs in the ICU.

Communication between nurses and their patients in the ICU is also often adversely affected by the therapeutic environment, such as patient emergencies and the use of mechanical ventilation [27,28]. Mechanical ventilators are one of the greatest obstacles to communication. Although they are essential for critically-ill patients who are incapable of spontaneous breathing, they affect their ability to speak [29]; therefore, these patients need to employ other strategies for communication, such as using facial expressions and lip movements, which make communication extremely difficult [27,30]. Our participants strived to understand the needs of critically-ill patients through verbal and nonverbal communication, such as writing and body language. However, when the intentions were not conveyed properly, some patients responded aggressively, hindering respiratory treatment and ultimately prolonging treatment. This is in line with many previous findings [29,31,32] indicating that patients’ failure to effectively express their needs to nurses or their family members triggers negative emotions. In addition, participants had trouble interacting with caregivers who were extremely tense and sensitive. According to Lee and Yi [17], families of critically-ill patients experience fear and anxiety regarding the patients’ health state and strive to save the patient. Thus, nurses must consider this when addressing vulnerable patients and their families and must actively identify and resolve causes of discomfort in patients on mechanical ventilation (i.e., by using appropriate analgesics/sedatives and removing the ventilator). Further, considering a systematic review revealing that electronic communication devices enable efficient communication with critically-ill patients through touch or eye blinks [33], Korea should also keep abreast with technological advances in communication technology.

Concerning theme 2, as participants experienced emotional exhaustion from being misunderstood or unfairly criticized by patients and their families, they contemplated and doubted the occupational values of nursing. Park and Lee [7] found that higher job satisfaction for ICU nurses is associated with better communication. This is consistent with our participants’ doubt for choosing the nursing profession. However, instead of giving up on this profession, they closely observed the effective communication skills of more experienced nurses, actively learned about therapeutic communication through books and videos, and applied their learnings during practice. Similar results were reported by Park and Oh [3] that patient-centered communication competency among critical care nurses was the highest when a biopsychosocial perspective, focused on delivery of factual information, was followed and the lowest in the therapeutic alliance domain, which is required for performing cooperative care with patients. Therapeutic communication provided by nurses to patients and their families in the ICU effectively diminished their psychological burden and fostered positive responses from families [34]. Currently, ICUs implement a systematic education system for nurses that focuses on therapeutic techniques, such as hemodynamic monitoring, mechanical ventilation care, aspiration, and extracorporeal membrane oxygenation; however, they lack a program targeting effective therapeutic communication with patients and caregivers. The communication difficulties experienced by nurses will persist without this additional program; thus, its implementation is critical to improve patient satisfaction and nursing quality of care. Further, instead of coercing unilateral effort from critical care nurses, nurse managers should pay attention to nurses’ emotional wellbeing and promptly develop systems to offset potential burnout, such as voluntary counseling systems or measures to “refresh” nurses.

Concerning theme 3, participants learned that communication is a challenging but essential aspect of critical care. The concept of communication resonates through Travelbee’s model [24,25]. Getting to know another human being is as important as performing procedures. A nurse must establish a rapport with the patient and the patient’s caregivers, otherwise he or she will not know the patient’s needs. As a place where life-and-death decisions are made, the ICU induces anxiety in critically-ill patients and their caregivers. Hence, nurses should fully empathize with patients and their caregivers [4,5,17].

Travelbee [24,25] emphasized the relationship between the nurse and the patient by establishing the Human-to-Human relationship model, which gives meaning to disease and suffering based on empathy, compassion, and rapport building. In addition, it presents concepts, such as disease, hope, human-to-human relations, communication, interaction, patient’s needs, perception, pain, finding meaning, therapeutic use of communication, and self-actualization. The participants cultivated empathy and active listening skills when speaking with patients and their families, and, as they spend more time doing so, their quality of care and nonverbal communication skills (such as eye contact, soft touch, and tears) improve and became more genuine. Our findings are consistent with the meaning of human-centered care suggested by Jang and Kim [35], which involves paying close attention to and protecting patients’ lives, deeply empathizing with patients from a humanistic perspective, and being sincere. The experience of nursing, including active interaction, has a positive impact on establishing the roles and caring attitudes of professional nurses [36], which is significant for critical care nurses. Patient-family-centered care, which has been confirmed to positively promote critically-ill patients’ recovery worldwide [1], is possible when nurses engage in therapeutic communication with patients and their families through dynamic interactions [34,37]. Therefore, critical care nurses and nurse managers should pay attention to communication and develop an effective communication course that can be applied in clinical practice. To do this, first, it is necessary to hire appropriate nursing personnel for active therapeutic communication with the patients and their families in an ICU. Second, continuous, and diverse educational opportunities should be provided to critical care nurses, along with policy strategies. For example, at the organizational level, it is necessary to develop manuals on how to deal with difficult situations by gathering challenging communication cases from actual clinical practice. Simulation education for communication is an important component of the nursing curriculum.

### Limitations

First, this study included a small number of participants; however, we ensured that the maximum data was collected from these participants. Second, specific information was collected from only those nurses who provided direct care in the ICU of a general hospital in a large city in Korea. The homogeneity and dynamics of the focus groups may have resulted in congruent opinions. Third, because the experiences of nurses from only one hospital were analyzed, caution should be exercised in generalizing our results and applying them to other hospitals in Korea. Therefore, follow-up studies with larger sample sizes and more representative participants are warranted.

## Conclusion

This qualitative study explored critical care nurses’ communication skills and experiences with patients and caregivers from various perspectives. Although these nurses felt discouraged by the unexpected communication difficulties with patients and their families, they recognized that they could address these difficulties by improving their communication skills over time through experience and learning. They realized that empathy, active listening, and physical interaction with patients and their families enabled meaningful communication and have gradually learned that effective communication is an indispensable tool in providing nursing care to critically-ill patients.

## Supporting information

S1 File COREQ checklist and coding tree. (DOCX)

## References

[1] Mitchell ML, Coyer F, Kean S, Stone R, Murfield J, Dwan T. Patient, family-centred care interventions within the adult ICU setting: an integrative review. Aust Crit Care. 2016; 29:179–193. 10.1016/j.aucc.2016.08.002 27592540

[2] Happ MB, Garrett K, Thomas DD, Tate J, George E, Houze M, et al Nurse-patient communication interactions in the intensive care unit. Am J Crit Care. 2011; 20:e28–40. 10.4037/ajcc2011433 21362711 PMC3222584

[3] Park Y, Oh EG. Factors related to intensive care unit nurses’ patient centered communication competency. J Korean Crit Care Nurs. 2018; 11:51–62.

[4] Ahn JW, Kim KS. ICU nurses' perceptions of communication difficulties, importance, satisfaction and communication barrier with patient families. Perspect Nurs Sci. 2013; 10:12–23.

[5] Webster D. Using standardized patients to teach therapeutic communication in psychiatric nursing. Clin Simul Nurs. 2014; 10(2):e81–e86.

[6] Webster D. Promoting therapeutic communication and patient-centered care using standardized patients. J Nurs Educ. 2013; 52:645–648. 10.3928/01484834-20131014-06 24127180

[7] Park EJ, Lee YM. Effect of professional autonomy, communication satisfaction, and resilience on the job satisfaction of intensive care unit nurses. J Korean Crit Care Nurs. 2018; 11:63–74.

[8] Ali M. Communication skills 1: benefits of effective communication for patients. Nurs Times. 2017; 113:18–19.

[9] McKinley CJ, Perino C. Examining communication competence as a contributing factor in health care workers’ job satisfaction and tendency to report errors. J Commun Healthc. 2013; 6:158–165.

[10] Wright KB, Banas JA, Bessarabova E, Bernard DR. A communication competence approach to examining health care social support, stress, and job burnout. Health Commun. 2010; 25:375–382. 10.1080/10410231003775206 20512719

[11] Radtke K. Improving patient satisfaction with nursing communication using bedside shift report. Clin Nurse Spec. 2013; 27:19–25. 10.1097/NUR.0b013e3182777011 23222024

[12] Abdelhadi N, Drach-Zahavy A. Promoting patient care: work engagement as a mediator between ward service climate and patient-centred care. J Adv Nurs. 2012; 68:1276–1287. 10.1111/j.1365-2648.2011.05834.x 21913960

[13] Boykins AD. Core communication competencies in patient-centered care. ABNF J. 2014; 25:40–45. 24855804

[14] Im SI, Park J, Kim HS. The effects of nurse’s communication and self-leadership on nursing performance. Korean J Occupat Health Nurs. 2012; 21:274–282.

[15] Lee HS, Kim JK. Relationship among communication competence, communication types, and organizational commitment in hospital nurses. J Korean Acad Nurs Admin. 2010; 16:488–496.

[16] Loghmani L, Borhani F, Abbaszadeh A. Factors affecting the nurse-patients' family communication in intensive care unit of kerman: a qualitative study. J Caring Sci. 2014; 3:67–82. 10.5681/jcs.2014.008 25276750 PMC4134163

[17] Lee M, Yi M. Experiences of families in the intensive care unit: interactions with health care providers. Korean J Adult Nurs. 2017; 29:76–86.

[18] Slatore CG, Hansen L, Ganzini L, Press N, Osborne ML, Chestnutt MS, et al Communication by nurses in the intensive care unit: qualitative analysis of domains of patient-centered care. Am J Crit Care. 2012; 21:410–418. 10.4037/ajcc2012124 23117904 PMC3992836

[19] Tong A, Sainsbury P, Craig J. Consolidated criteria for reporting qualitative research (COREQ): A 32-item checklist for interviews and focus groups. Int J Qual Health Care. 2007; 19:349–357. 10.1093/intqhc/mzm042 17872937

[20] Morgan DL. Focus groups. Ann Rev Sociol. 1996; 22:129–152.

[21] Krueger RA, Casey MA. Focus group interviewing Handbook of practical program evaluation 3rd edition. San Francisco (CA): Jossey-Bass; 2010.

[22] Colaizzi PF. Psychological research as the phenomenologist views it In: Valle R, King M, editors. Existential-phenomenological alternatives for psychology. New York, NY: Oxford University Press; 1978.

[23] Sandelowski M. The problem of rigor in qualitative research. Adv Nurs Sci. 1986; 8:27–37. 10.1097/00012272-198604000-00005 3083765

[24] Travelbee J. What’s wrong with sympathy? Am J Nurs. 1964; 64:68–71. 14113741

[25] Travelbee J. Interpersonal aspects of nursing 2nd edition. Philadelphia (PA): F. A. Davis Company; 1971.

[26] Kang J, Cho YS, Jeong YJ, Kim SG, Yun S, Shim M. Development and validation of a measurement to assess person-centered critical care nursing. J Korean Acad Nurs. 2018; 48:323–334. 10.4040/jkan.2018.48.3.323 29968688

[27] Foà C, Cavalli L, Maltoni A, Tosello N, Sangilles C, Maron I, et al Communications and relationships between patient and nurse in intensive care unit: knowledge, knowledge of the work, knowledge of the emotional state. Acta Bio-med. 2016; 87:71–82. 27874846

[28] Azoulay E, Herridge M. Understanding ICU staff burnout: the show must go on. Am J Respirat Crit Care Med. 2011; 184:1099–1100. 22086984 10.1164/rccm.201109-1638ED

[29] Dithole K, Sibanda S, Moleki MM, Thupayagale-Tshweneagae G. Exploring communication challenges between nurses and mechanically ventilated patients in the intensive care unit: a structured review. Worldviews Evid Based Nurs. 2016; 13:197–206. 10.1111/wvn.12146 26860230

[30] Martinho CI, Rodrigues IT. Communication of mechanically ventilated patients in intensive care units. Rev Bras Ter Intensiva. 2016; 28:132–140. 10.5935/0103-507X.20160027 27410408 PMC4943050

[31] Song YG, Yun EK. Model for unplanned self extubation of ICU patients using system dynamics approach. J Korean Acad Nurs. 2015; 45:280–292. 10.4040/jkan.2015.45.2.280 25947190

[32] Samuelson KA. Adult intensive care patients' perception of endotracheal tube-related discomforts: a prospective evaluation. Heart Lung. 2011; 40:49–55. 10.1016/j.hrtlng.2009.12.009 20561863

[33] Ten Hoorn S, Elbers PW, Girbes AR, Tuinman PR. Communicating with conscious and mechanically ventilated critically ill patients: a systematic review. Crit Care. 2016; 20:333 10.1186/s13054-016-1483-2 27756433 PMC5070186

[34] Adams A, Mannix T, Harrington A. Nurses' communication with families in the intensive care unit—a literature review. Nurs Crit Care. 2017; 22:70–80. 10.1111/nicc.12141 25583405

[35] Jang MS, Kim S. Person-centered relational care experienced by critical care nurses: an interpretative phenomenological analysis study. J Korean Acad Nurs. 2019; 49:423–436. 10.4040/jkan.2019.49.4.423 31477672

[36] Chen C, Chow AYM, Tang S. Bereavement process of professional caregivers after deaths of their patients: a meta-ethnographic synthesis of qualitative studies and an integrated model. Int J Nurs Stud. 2018; 88:104–113. 10.1016/j.ijnurstu.2018.08.010 30227279

[37] Chiara G, Lucia G. The patient in intensive care: communication with the critical patient and his family members: a narrative review. Nurs Healthc Int J. 2018; 2:000134.

