# Supplementary material for: Critical care nurses’ communication experiences with patients and families in an intensive care unit: A qualitative study
Source: PLoS One. 2020 Jul 9;15(7):e0235694. doi: 10.1371/journal.pone.0235694 (PMC7347110; doi:10.1371/journal.pone.0235694)
Supplement: S1 File — (DOCX) [file pone.0235694.s001.docx]

**Supplementary information 1: COREQ checklist (32items)**

| **Domain 1: Research team and reflexivity** | **Answer** | **Location in manuscript (Section, page no.)** |
| --- | --- | --- |
| **Personal Characteristics** |  |  |
| 1. Interviewer/facilitator  Which author/s conducted the interview or focus group? | Yoo & Shim | Materials and methods,  page 7 |
| 2. Credentials  What were the researcher’s credentials? | Yoo & Shim (PhD, RN)  Lim (MSN, RN) | Title page |
| 3. Occupation  What was their occupation at the time of the study? | Yes | Materials and methods,  page 6 |
| 4. Gender  Was the researcher male or female | Yes | Materials and methods,  page 8 |
| 5. Experience and training  What experience or training did the researcher have? | Yes | Materials and methods,  page 6 |
| **Relationship with participants** |  |  |
| 6. Relationship established  Was a relationship established prior to study commencement? | Yes | - |
| 7. Participant knowledge of the interviewer  What did the participants know about the researcher? e.g. personal goals, reasons for doing the research | Participants were briefed on the purpose of the study and understood that it was a research. Educational ethical approval had been granted, participants reviewed the participant information documentation prior to giving their written informed consent to be involved. | Materials and methods,  page 9 |
| 8. Interviewer characteristics  What characteristics were reported about the interviewer/facilitator? e.g. Bias, assumptions, reasons and interests in the research topic | No other interviewer related biases identified. | - |
| **Domain 2: study design** |  |  |
| **Theoretical framework** |  |  |
| 9. Methodological orientation and Theory  What methodological orientation was stated to underpin the study?  e.g. grounded theory, discourse analysis, ethnography, phenomenology, content analysis | Phenomenology | Materials and methods,  page 9 |
| **Participant selection** |  |  |
| 10. Sampling  How were participants selected?  e.g. purposive, convenience, consecutive, snowball | Snowball sampling | Materials and methods,  page 5 |
| 11. Method of approach  How were participants approached?  e.g. face-to-face, telephone, mail, email | Face to face | Materials and methods,  page 6 |
| 12. Sample size  How many participants were in the study | 16 | Results, 16 |
| 13. Non-participation  How many people refused to participate or dropped out? Reasons? | Of the 16 respondents that were invited for a semi-structured interview, all gave informed consent and completed the interview. There were no participants who subsequently refused to participate, withdrew consent or dropped out. | Materials and methods,  page 5 |
| **Setting** |  |  |
| 14. Setting of data collection  Where was the data collected? e.g. home, clinic, workplace | The FGIs were moderated by the principal investigator and were conducted in a quiet conference room | Materials and methods,  page 8 |
| 15. Presence of non-participants  Was anyone else present besides the participants and researchers? | No | - |
| 16. Description of sample  What are the important characteristics of the sample? e.g. demographic data, date | All participants were women (mean age = 29.0 years; mean clinical career = 4.5 years). | Table 1, 5  page 5-6 |
| **Data collection** |  |  |
| 17. Interview guide  Were questions, prompts, guides provided by the authors? Was it pilot tested? | For data collection, the interview questions were structured according to guidelines developed for focus-group methodology | Materials and methods,  page 7-8  Table 2 |
| 18. Repeat interviews  Were repeat interviews carried out?  If yes, how many | No | - |
| 19. Audio/visual recording  Did the research use audio or visual recording to collect the data? | FGIs were audio-recorded with participants’ consent, and the recordings were transcribed and analyzed immediately after the each FGI. | Materials and methods,  page 8 |
| 20. Field notes  Were field notes made during and/or after the interview or focus group? | One assistant helped in facilitating the interviews and took notes. | Materials and methods, page 8 |
| 21. Duration  What was the duration of the interviews or focus group? | The duration of each interview was about 60–90 minutes | Materials and methods, page 8 |
| 22. Data saturation  Was data saturation discussed? | the data were collected and analyzed until saturation was achieved (i.e., no new content emerged). | Materials and methods, page 10 |
| 23. Transcripts returned  Were transcripts returned to participants for comment and/or correction? | To ensure auditability, the raw data for the identified themes were presented in the results, such that the readers could understand the decision-making process. | Materials and methods, page 10 |
| **Domain 3: analysis and findings** |  |  |
| **Data analysis** |  |  |
| 24. Number of data coders  How many data coders coded the data? | One | - |
| 25. Description of the coding tree  Did authors provide a description of the coding tree? | Extraction of themes from relevant meanings and development of a coding tree, with the meanings organized into themes | Materials and methods,  page 9 |
| 26. Derivation of themes  Were themes identified in advance or derived from the data | Searching for other interpretations of participant statements using various contexts; Extraction of themes from relevant meanings and development of a coding tree, with the meanings organized into themes | Materials and methods, page 9 |
| 27. Software  What software, if applicable, was used to manage the data? | Microsoft Word and Excel | - |
| 28. Participant checking  Did participants provide feedback on the findings? | ~the data verified by three participants to establish the universality and validity of the identified themes | Materials and methods, page 10 |
| **Reporting** |  |  |
| 29. Quotations presented  Were participant quotations presented to illustrate the themes/findings?  Was each quotation identified?  e.g. participant number | Yes, specific comments were supported with direct quotes attributed to anonymised participant. | Results,  page 17-24 |
| 30. Data and findings consistent  Was there consistency between the data presented and the findings? | Yes | Results,  page 11-24 |
| 31. Clarity of major themes  Were major themes clearly presented in the findings? | Yes | Results,  page 11-24 |
| 32. Clarity of minor themes  Is there a description of diverse cases or discussion of minor themes? | Yes | Results,  page 11-24 |

**Supplementary information 2: Coding tree**

Being essential for care

Expressing warmth and respect

Mediator

Active listening

Empathy

Knowhow learned through persistent effort

Finding out better communication style

Nurse-related factor

Patient- and family-related factor

System-related factor

Doubts about the nursing profession

Approaches to the

patient-centered nursing

Learning through trial and error

Unexpected communication difficulties
